# Utilization, quality, and spending for pediatric Medicaid enrollees with primary care in health centers vs non-health centers

**DOI:** 10.1186/s12887-024-04547-y

**Published:** 2024-02-08

**Authors:** Anna Volerman, Bradley Carlson, Wen Wan, Manoradhan Murugesan, Nour Asfour, Joshua Bolton, Marshall H. Chin, Alek Sripipatana, Robert S. Nocon

**Affiliations:** 1 Departments of Medicine and Pediatrics, University of Chicago, 5841 S Maryland Ave, Chicago, IL 60637 USA; 2 University of Chicago Pritzker School of Medicine, 924 E 57th St, Chicago, IL 60637 USA; 3 Department of Medicine, University of Chicago, 5841 S Maryland Ave, Chicago, IL 60637 USA; 4 Department of Public Health Sciences, University of Chicago, 5841 S Maryland Ave, Chicago, IL 60637 USA; 5 Health Resources and Services Administration (Affiliation at Time Research Conducted), 5600 Fishers Lane, Rockville, MD 20857 USA; 6 Health Resources and Services Administration, 5600 Fishers Lane, Rockville, MD 20857 USA; 7 Department of Health Systems Science, Kaiser Permanente Bernard J. Tyson School of Medicine, 98 S Los Robles Ave, Pasadena, CA 91101 USA

**Keywords:** Children, Health centers, Medicaid, Primary care

## Abstract

**Background:**

Limited research has explored the performance of health centers (HCs) compared to other primary care settings among children in the United States. We evaluated utilization, quality, and expenditures for pediatric Medicaid enrollees receiving care in HCs versus non-HCs.

**Methods:**

This national cross-sectional study utilized 2012 Medicaid Analytic eXtract (MAX) claims to examine children 0–17 years with a primary care visit, stratified by whether majority (> 50%) of primary care visits were at HCs or non-HCs. Outcome measures include utilization (primary care visits, non-primary care outpatient visits, prescription claims, Emergency Department (ED) visits, hospitalizations) and quality (well-child visits, avoidable ED visits, avoidable hospitalizations). For children enrolled in fee-for-service Medicaid, we also measured expenditures. Propensity score-based overlap weighting was used to balance covariates.

**Results:**

A total of 2,383,270 Medicaid-enrolled children received the majority of their primary care at HCs, while 18,540,743 did at non-HCs. In adjusted analyses, HC patients had 20% more primary care visits, 15% less non-primary care outpatient visits, and 21% less prescription claims than non-HC patients. ED visits were similar across the two groups, while HC patients had 7% lower chance of hospitalization than non-HC. Quality of care outcomes favored HC patients in main analyses, but results were less robust when excluding managed care beneficiaries. Total expenditures among the fee-for-service subpopulation were lower by $239 (8%) for HC patients.

**Conclusions:**

In this study of nationwide claims data to evaluate healthcare utilization, quality, and spending among Medicaid-enrolled children who receive primary care at HCs versus non-HCs, findings suggest primary care delivery in HCs may be associated with a more cost-effective model of healthcare for children.

**Supplementary Information:**

The online version contains supplementary material available at 10.1186/s12887-024-04547-y.

## Background

Health centers (HCs) provide comprehensive health care services to over nine million United States children [1, 2]. HCs are health care organizations that receive federal support to deliver care to underserved populations regardless of ability to pay. The majority receive grant funding from the Health Resources and Services Administration (HRSA) through Sect. 330 of the Public Health Service Act as well as enhanced Medicaid reimbursement rates [3]. Nearly 75% of children seen at HCs are enrolled in Medicaid or the Children’s Health Insurance Program, which are public health insurance programs for people with low-income in the United States [4, 5].

Over the past several decades, the role of HCs has expanded due to increased funding from the growth of Medicaid coverage, Affordable Care Act, and Sect. 330 Act appropriations [6–8]. These investments have enabled an expansion of children served, including a nearly 50% increase in the proportion of children seen at HCs over 10 years [9].

Despite substantial growth of pediatric care provided by HCs, limited research has evaluated the relative performance and value of HCs compared to other primary care settings for children. Studies suggest children receiving primary care at HCs have fewer emergency department (ED) visits, [10] lower total healthcare expenditures, [4, 11] and similar or better quality of care [12] as compared to other settings. However, this research primarily utilizes surveys to assess utilization and spending outcomes or examines claims data for only a few states.

This study moves beyond state-specific and survey-based studies to compare the care of children in Medicaid seen at HCs versus non-HCs using nationwide claims data. We evaluate utilization, quality, and spending by pediatric Medicaid enrollees using a national dataset to present a more comprehensive understanding of the benefits provided by HCs to children.

## Methods

This cross-sectional study examined the association between primary care setting and healthcare utilization, quality, and spending outcomes among Medicaid-enrolled children in the 50 states and District of Columbia in 2012. University of Chicago’s Institutional Review Board approved this study.

### Data Collection

**Fig. 1:**
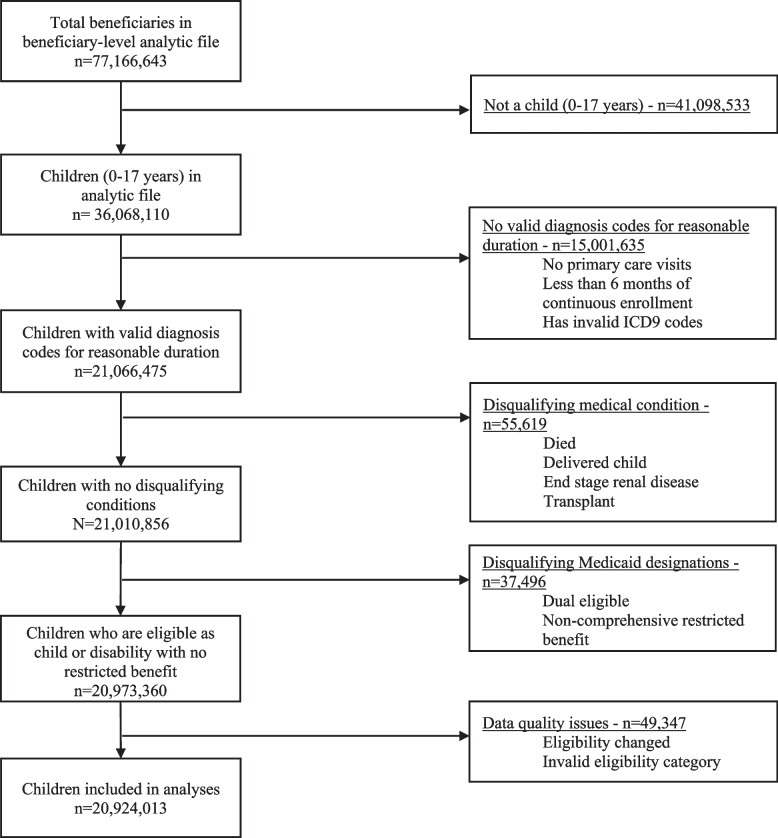
Exclusions

We obtained claims from the Medicaid Analytic eXtract (MAX) files [13] and constructed an analytic dataset of children 0–17 years who were enrolled in Medicaid for a reasonable duration and used ambulatory primary care services in 2012. All dental, transportation, and long-term care claims were excluded. Children were excluded if they died, delivered a baby, or had conditions qualifying them for Medicare (e.g., end-stage renal disease, transplant) during that year (full list of study exclusion criteria in Fig. 1; online). These exclusions were made to focus our analyses on medical services covered by Medicaid without other insurance plans (e.g., Medicare, dental) and to ensure the analyses included sufficient duration of enrollment to adequately characterize service utilization.

Our utilization outcomes were number of primary care visits, non-primary outpatient care (e.g., subspecialty care, behavioral health), prescription drug claims, ED visits, and inpatient admissions. For spending, we calculated the sum of total payments from Medicaid and third-party payers. Expenditures outcome variables were constructed for each category of utilization and total amount.

We examined claim-based quality metrics for children. After a review of the Core Set of Children’s Health Care Quality Measures for Medicaid and CHIP, [14] we focused on two sets of quality metrics based on the available data in MAX: well-child visits as well as avoidable or ambulatory care sensitive (ACS) emergency department visits and hospitalizations. Given the importance of preventive care for children, well-child visits were measured for children in two age groups with well-established quality metrics—3–6 and 12–17 years; children were included if they had no more than a one-month enrollment gap. For each age group, well-child visits were measured as the proportion of children who had at least one well-child visit with a primary care provider during the year. Also, since high-quality, accessible primary care is hypothesized to prevent some types of ED visits and hospitalizations, quality measures were included to identify ACS utilization. ACS ED visits were measured using an adaptation of the NYU/Billings algorithm for classifying ED utilization, [15, 16] and ACS hospital admissions were measured using the Agency for Healthcare Research and Quality Pediatric Quality Indicators [17].

**Fig. 2:**
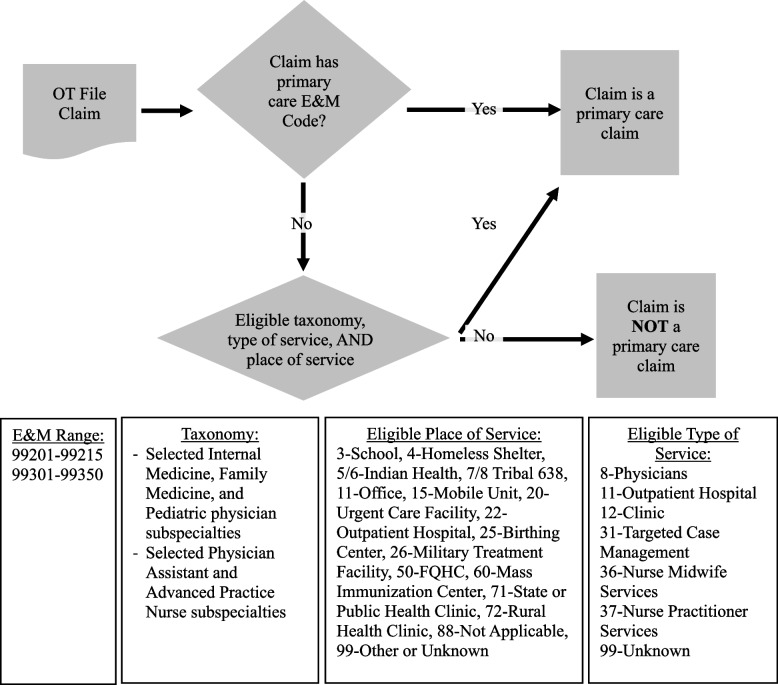
Flowchart representation of the identification of primary care claims

The main independent variable was whether the patient received the majority of their primary care visits at HCs. Primary care visits were identified using a combination of evaluation and management codes, provider taxonomies, and claim setting or type of service [18]. Flowchart representation of this assignment strategy is in Fig. 2; online; and specific codes are listed in Table 1. The setting for each primary care visit (HC or non-HC) was determined by examining national provider identifier, claim type, and place of service in each claim. To identify HC settings, we created a listing of HC identifiers from Medicare Cost Reports (HCRIS) and HRSA Uniform Data System (UDS) datasets. This list was then linked to the National Plan and Provider Enumeration System (NPPES) to obtain the national provider identifier number used in Medicaid claims. Linkages across the HCRIS, UDS, and NPPES were performed by direct match of common identifiers across datasets, or in some cases, location and clinic name-based matching using text-based matching algorithms. All non-exact text-based matches were manually reviewed and supplemented by comparison of public online information to confirm accuracy [19]. These identifiers, along with type of program and place of service code, helped us identify health center claims. We categorized health center patients as individuals with more than half of primary care visits occurring at a health center. Individuals with less than or equal to half of all primary care visits occurring at a health center or no primary care visits at a health center were categorized as non-health center patients (hospital outpatient, physician office, or a mix).

**Table 1:** Domains and variables

Variables by domain	Definition of variables
**Dependent variables**	
Utilization	Number of primary care visits
	Number of non-primary outpatient care (e.g., subspecialty care, behavioral health)
	Number of prescription drug claims
	Number of emergency department visits
	Number of inpatient admissions
Quality	Well-child visits for two age groups: 3–6 years old and 12–17 years old Assessed based on CPT codes and ICD codes: CPT codes: 99,381–99,384, 99,391–99,394 (CPT codes for well-child visits by age: 0–15 months = 99,381, 99,382, 99,391, 99,392; 3–6 years = 99,382, 99,383, 99,392, 99,393; 12–17 years = 99,384, 99,394) ICD codes: V202, V700, V703, V705, V706, V708, V709
	Avoidable or ambulatory care sensitive emergency department visits Measured using an adaptation of the NYU/Billings algorithm for classifying ED utilization [15, 16]
	Avoidable or ambulatory care sensitive hospital admissions Measured using the Agency for Healthcare Research and Quality Pediatric Quality Indicators [17]
Spending	Total amount calculated as a sum of payments from Medicaid and third-party payers
	Amount for each category of utilization calculated as a sum of payments from Medicaid and third-party payers
**Independent variable**	
Health center or non-health center patient	Health center patients = individuals with more than half of primary care visits occurring at a health center* Non-health center patients = individuals with less than or equal to half of all primary care visits occurring at a health center or no primary care visits at a health center* Primary care visits Identifying using provider specialty and procedure codes CPT codes: 99,201–99205, 99,211–99,215, 99,381–99,387, 99,391–99,397 ICD codes: any Health center setting Determined using national provider identifier, claim type, and place of service in each claim

*Abbreviations*: *CPT* Current Procedural Terminology, *ICD9* International Classification of Disease, Ninth Revision

^*^Sensitivity analysis examined children who received 100% of primary care visits at health centers versus 100% at non-health center

We considered covariates that were potentially related to the primary care setting and/or influenced healthcare utilization, quality, and spending. Covariates included patient demographics (age, race/ethnicity, gender), location (U.S. state, rural versus urban, distance to closest HC), insurance characteristics (eligibility category, months of eligibility, Temporary Aid for Needy Families (TANF) program indicator), and disease burden. Geographic variables included: patient residence ZIP code in rural versus urban area based on USDA Rural Urban Commuting Area codes [20] and distance from patient residence ZIP code (using centroid of ZIP code) to the closest HC site. To measure disease burden, the Chronic Illness and Disability Payment System for Medicaid with the Medicaid Rx model (CDPS + Rx) was applied [21]. The CDPS + Rx utilizes information from inpatient and outpatient diagnosis codes, as well as filled prescription medications to identify categories of disease associated with high healthcare resource use. The CDPS + Rx produces a continuous risk score indexed to 1.00, which reflects an average predicted expenditure level. A CDPS + Rx score of 1.1 would indicate an individual with at 10% higher predicted expenditure, based on diagnoses and prescriptions reflected in claims [22, 23].

The primary analysis included all 2012 pediatric Medicaid enrollees (fee-for-service or managed care) to analyze utilization and quality. A sensitivity analysis was conducted focusing on children nationwide who were enrolled in fee-for-service Medicaid only. While a majority of children are in Medicaid managed care plans, [24] this fee-for-service analysis allowed us to examine spending, which is not included in MAX dataset for children enrolled in managed care Medicaid [13]. Further, past studies have questioned the quality of managed care data in some states, [25] and this sensitivity analysis allows for results not subject to data quality concerns. An additional sensitivity analysis was conducted that compared children who had 100% of their primary care visits at HCs and children who had 100% of their primary care visits at non-HCs.

### Statistical Analyses

Descriptive analyses were conducted to summarize patient characteristics by primary care setting. We used propensity score overlap weights (OW) [26] to construct balanced groups of HC and non-HC patients. OW has been shown to achieve desirable covariate balance and precision, while allowing for inclusion of the entire study population in final weighted analyses [26, 27]. We calculated propensity scores using logistic regression to model the probability of treatment assigned to a HC based on the covariates. We evaluated the balance of covariates by calculating standardized mean differences with and without OW weights (Table 2).

**Table 2:** Balancing table

Characteristics / Covariates used for weighting	Crude					Overlap weight method				
	**Health Center *n* = 2,379,417**	**Non-Health Center *n* = 18,507,232**	**Mean difference**	**Standard error of mean difference**	**Standardized difference**	**Health Center *n* = 2,379,417**	**Non-Health Center *n* = 18,507,232**	**Mean difference**	**Standard error of mean difference**	**Standardized difference**
	**No. (%) or mean (SD)**	**No. (%) or mean (SD)**				**No. (%) or mean (SD)**	**No. (%) or mean (SD)**			
Female, # (%)	1,176,939 (49.39)	9,074,068 (48.95)	0.44	0.5	0.88	927,684 (49.29)	927,684 (49.29)	0	0.5	0
Age, mean (SD)	7.86 (5.02)	7.78 (5.00)	0.08	5.01	1.54	7.8 (4.47)	7.8 (1.6)	0	3.36	0
Race/ethnicity, # (%)										
White	554,656 (23.27)	7,159,942 (38.62)	-15.34	0.46	33.66	476,232 (25.30)	476,232 (25.30)	0	0.43	0
Black	477,466 (20.03)	3,951,305 (21.31)	-1.28	0.4	3.15	385,242 (20.47)	385,242 (20.47)	0	0.4	0
American Indian or Alaska Native	32,695 (1.37)	229,922 (1.24)	0.13	0.11	1.16	27,004 (1.43)	27,004 (1.43)	0	0.12	0
Asian	60,817 (2.55)	447,471 (2.41)	0.14	0.16	0.89	49,599 (2.64)	49,599 (2.64)	0	0.16	0
Hispanic/Latino or Hispanic and > 1 race	971,934 (40.78)	4,986,916 (26.90)	13.88	0.47	29.66	726,751 (38.61)	726,751 (38.61)	0	0.49	0
Native Hawaiian or other Pacific Islander	20,875 (0.88)	87,280 (0.47)	0.41	0.08	4.96	15,417 (0.82)	15,417 (0.82)	0	0.09	0
Non-Hispanic and > 1 race	13,354 (0.56)	150,640 (0.81)	-0.25	0.08	3.05	11,462 (0.61)	11,462 (0.61)	0	0.08	0
Unknown/Missing	251,473 (10.55)	1,527,267 (8.24)	2.31	0.29	7.94	190,418 (10.12)	190,418 (10.12)	0	0.3	0
Urban, # (%)	2,001,281 (84.04)	14,887,775 (80.35)	3.69	0.38	9.66	1,587,170 (84.33)	1,587,170 (84.33)	0	0.36	0
Distance to nearest health center, kilometers, mean (SD)	14.92 (20.86)	22.30 (25.54)	-7.38	23.32	31.67	15.63 (19.46)	15.63 (5.75)	0	14.35	0
Medicaid eligibility, # (%)										
Child	2,296,298 (96.35)	17,576,244 (94.80)	1.55	0.21	7.56	1,808,518 (96.09)	1,808,518 (96.09)	0	0.19	0
Disabled	84,428 (3.54)	911,120 (4.91)	-1.37	0.2	6.82	71,557 (3.80)	71,557 (3.80)	0	0.19	0
Demonstration projects	2,544 (0.11)	53,379 (0.29)	-0.18	0.04	4.08	2,051 (0.11)	2,051 (0.11)	0	0.03	0
Temporary Assistance for Needy Families (TANF) eligible, # (%)	425,187 (17.84)	1,895,533 (10.22)	7.62	0.35	22.06	315,761 (16.78)	315,761 (16.78)	0	0.37	0
Eligible months, mean (SD)										
Total	11.40 (1.43)	11.38 (1.46)	0.02	1.44	1.71	11.41 (1.27)	11.41 (0.46)	0	0.95	0
Managed care	8.07 (4.97)	8.57 (4.67)	-0.5	4.83	10.34	8.04 (4.43)	8.04 (1.60)	0	3.33	0
Chronic Disease Payment System Rx score, mean (SD)	0.99 (1.77)	1.17 (2.32)	-0.18	2.07	8.65	1.02 (1.64)	1.02 (0.55)	0	1.22	0
State, # (%)										
AK	5645 (0.2)	49,445 (0.3)	-0.03	0.05	0.6	5032 (0.3)	5032 (0.3)	0	0.05	0
AL	30,598 (1.3)	369,129 (2)	-0.71	0.13	5.57	27,611 (1.5)	27,611 (1.5)	0	0.12	0
AR	16 (0)	1399 (0)	-0.01	0.01	1.07	16 (0)	16 (0)	0	0	0
AZ	34,037 (1.4)	464,327 (2.5)	-1.08	0.14	7.76	31,338 (1.7)	31,338 (1.7)	0	0.13	0
CA	637,844 (26.8)	1,831,440 (9.9)	16.89	0.38	44.73	467,866 (24.9)	467,866 (24.9)	0	0.43	0
CO	65,994 (2.8)	178,492 (1)	1.81	0.14	13.38	47,504 (2.5)	47,504 (2.5)	0	0.16	0
CT	52,070 (2.2)	150,110 (0.8)	1.38	0.12	11.34	37,005 (2)	37,005 (2)	0	0.14	0
DC	13,698 (0.6)	46,143 (0.2)	0.33	0.06	5.09	10,497 (0.6)	10,497 (0.6)	0	0.07	0
DE	262 (0)	71,155 (0.4)	-0.37	0.04	8.41	261 (0)	261 (0)	0	0.01	0
FL	66,818 (2.8)	1,106,803 (6)	-3.17	0.2	15.51	62,682 (3.3)	62,682 (3.3)	0	0.18	0
GA	26,286 (1.1)	666,914 (3.6)	-2.49	0.15	16.52	25,025 (1.3)	25,025 (1.3)	0	0.11	0
HI	7203 (0.3)	57,224 (0.3)	-0.01	0.06	0.12	6319 (0.3)	6319 (0.3)	0	0.06	0
IA	19,846 (0.8)	174,461 (0.9)	-0.11	0.09	1.15	17,269 (0.9)	17,269 (0.9)	0	0.1	0
ID	9020 (0.4)	109,775 (0.6)	-0.21	0.07	3.07	8245 (0.4)	8245 (0.4)	0	0.07	0
IL	187,651 (7.9)	845,569 (4.6)	3.31	0.24	13.75	148,754 (7.9)	148,754 (7.9)	0	0.27	0
IN	51,311 (2.2)	415,390 (2.2)	-0.09	0.15	0.6	44,875 (2.4)	44,875 (2.4)	0	0.15	0
KS	6779 (0.3)	160,384 (0.9)	-0.58	0.08	7.69	6423 (0.3)	6423 (0.3)	0	0.06	0
KY	16,181 (0.7)	355,569 (1.9)	-1.24	0.11	10.96	15,333 (0.8)	15,333 (0.8)	0	0.09	0
LA	22,713 (1)	489,490 (2.6)	-1.69	0.13	12.73	21,565 (1.1)	21,565 (1.1)	0	0.11	0
MA	34,278 (1.4)	343,301 (1.9)	-0.41	0.13	3.25	30,285 (1.6)	30,285 (1.6)	0	0.13	0
MD	50,574 (2.1)	34,977 (0.2)	1.93	0.11	18.17	19,844 (1.1)	19,844 (1.1)	0	0.1	0
ME	17,106 (0.7)	71,792 (0.4)	0.33	0.07	4.46	13,620 (0.7)	13,620 (0.7)	0	0.08	0
MI	57,672 (2.4)	666,954 (3.6)	-1.18	0.17	6.9	52,295 (2.8)	52,295 (2.8)	0	0.16	0
MN	1631 (0.1)	283,322 (1.5)	-1.46	0.09	16.46	1614 (0.1)	1614 (0.1)	0	0.03	0
MO	10,978 (0.5)	366,965 (2)	-1.52	0.11	13.87	10,596 (0.6)	10,596 (0.6)	0	0.07	0
MS	24,609 (1)	254,992 (1.4)	-0.34	0.11	3.14	21,973 (1.2)	21,973 (1.2)	0	0.11	0
MT	6092 (0.3)	47,993 (0.3)	0	0.05	0.06	5212 (0.3)	5212 (0.3)	0	0.05	0
NC	16,982 (0.7)	760,251 (4.1)	-3.39	0.15	22.24	16,519 (0.9)	16,519 (0.9)	0	0.09	0
ND	1681 (0.1)	24,016 (0.1)	-0.06	0.03	1.87	1524 (0.1)	1524 (0.1)	0	0.03	0
NE	4362 (0.2)	121,145 (0.7)	-0.47	0.06	7.29	4144 (0.2)	4144 (0.2)	0	0.05	0
NH	6365 (0.3)	65,268 (0.4)	-0.08	0.06	1.53	5721 (0.3)	5721 (0.3)	0	0.06	0
NJ	14,581 (0.6)	477,756 (2.6)	-1.96	0.12	15.74	14,076 (0.7)	14,076 (0.7)	0	0.09	0
NM	17,799 (0.7)	220,452 (1.2)	-0.44	0.1	4.52	16,323 (0.9)	16,323 (0.9)	0	0.09	0
NV	2739 (0.1)	40,595 (0.2)	-0.1	0.04	2.55	2498 (0.1)	2498 (0.1)	0	0.04	0
NY	198,985 (8.3)	1,232,610 (6.6)	1.7	0.26	6.46	168,058 (8.9)	168,058 (8.9)	0	0.29	0
OH	79,221 (3.3)	816,489 (4.4)	-1.08	0.19	5.6	71,519 (3.8)	71,519 (3.8)	0	0.19	0
OK	21,784 (0.9)	339,853 (1.8)	-0.92	0.12	7.9	20,152 (1.1)	20,152 (1.1)	0	0.1	0
OR	21,181 (0.9)	197,398 (1.1)	-0.18	0.1	1.79	18,801 (1)	18,801 (1)	0	0.1	0
PA	24,659 (1)	320,468 (1.7)	-0.69	0.12	5.95	22,582 (1.2)	22,582 (1.2)	0	0.11	0
RI	10,563 (0.4)	42,217 (0.2)	0.22	0.06	3.73	7979 (0.4)	7979 (0.4)	0	0.06	0
SC	49,257 (2.1)	364,546 (2)	0.1	0.14	0.72	42,403 (2.3)	42,403 (2.3)	0	0.15	0
SD	7104 (0.3)	45,624 (0.2)	0.05	0.05	1	6061 (0.3)	6061 (0.3)	0	0.06	0
TN	15,391 (0.6)	529,099 (2.9)	-2.21	0.13	16.9	14,919 (0.8)	14,919 (0.8)	0	0.09	0
TX	125,742 (5.3)	2,014,116 (10.9)	-5.59	0.27	20.62	117,713 (6.3)	117,713 (6.3)	0	0.24	0
UT	4266 (0.2)	127,038 (0.7)	-0.51	0.07	7.72	4082 (0.2)	4082 (0.2)	0	0.05	0
VA	9195 (0.4)	432,323 (2.3)	-1.95	0.12	16.87	8965 (0.5)	8965 (0.5)	0	0.07	0
VT	8365 (0.4)	40,515 (0.2)	0.13	0.05	2.49	6742 (0.4)	6742 (0.4)	0	0.06	0
WA	239,009 (10)	326,852 (1.8)	8.27	0.23	35.64	132,028 (7)	132,028 (7)	0	0.26	0
WI	37,576 (1.6)	326,383 (1.8)	-0.18	0.13	1.43	32,993 (1.8)	32,993 (1.8)	0	0.13	0
WV	9155 (0.4)	28,904 (0.2)	0.23	0.05	4.4	6881 (0.4)	6881 (0.4)	0	0.06	0
WY	396 (0)	33,310 (0.2)	-0.16	0.03	5.21	386 (0)	386 (0)	0	0.01	0

We performed generalized linear models (GLM) with the log link function and an appropriate distribution based on outcome type. For utilization and quality outcomes that were binary variables, we used a binomial distribution and estimated relative risk ratio (RR) with 95% confidence interval (CI), representing the risk of the event in the HC group divided by non-HC group. For utilization outcomes that were count variables, we used a negative binomial distribution to estimate means and their incidence rate ratio (IRR) with 95% CI, representing the ratio of the incidence rates between HC and non-HC groups. For spending outcomes, we used a gamma distribution to estimate means of payments and their IRR with 95% CI. We obtained crude (unadjusted) estimates of outcome variables for each group through models that did not incorporate weights and then adjusted estimates using OW. GLM models adjusted with OW did not include additional covariates. Analyses utilized SAS version 9.4 (Cary, NC).

## Results

In 2012, 2,383,270 Medicaid-enrolled children received the majority of their primary care at HCs, while 18,540,743 did so at non-HCs (Table 3).

**Table 3:** Characteristics of all children enrolled in Medicaid and children in fee-for-service only Medicaid in the United States in 2012, by primary care setting

**Characteristics**	**All Medicaid**		**Fee-for-service only Medicaid**	
	**Health Center**	**Non-Health Center**	**Health Center**	**Non-Health Center**
	**(*n* = 2,383,270)**	**(*n* = 18,540,743)**	**(*n* = 557,912)**	**(*n* = 3,530,021)**
	**No. (%) or mean (SD)**	**No. (%) or mean (SD)**	**No. (%) or mean (SD)**	**No. (%) or mean (SD)**
Female, # (%)	1,176,939 (49.39)	9,074,068 (48.95)	274,498 (49.22)	1,701,558 (48.23)
Age category, # (%)				
0–15 months	177,486 (7.45)	1,396,598 (7.53)	38,637 (6.93)	217,796 (6.17)
16 months-2 years	326,971 (13.72)	2,597,653 (14.01)	71,219 (12.77)	436,491 (12.37)
3–6 years	524,278 (22.00)	4,101,463 (22.12)	117,323 (21.03)	733,656 (20.78)
7–11 years	758,850 (31.84)	5,915,859 (31.91)	177,025 (31.73)	1,168,935 (33.11)
12–17 years	595,685 (25.00)	4,529,170 (24.43)	153,708 (27.55)	973,143 (27.57)
Race/ethnicity, # (%)				
White	554,656 (23.27)	7,159,942 (38.62)	147,966 (26.52)	1,515,561 (42.93)
Black	477,466 (20.03)	3,951,305 (21.31)	124,561 (22.33)	694,665 (19.68)
American Indian or Alaska Native	32,695 (1.37)	229,922 (1.24)	18,812 (3.37)	101,815 (2.88)
Asian	60,817 (2.55)	447,471 (2.41)	11,013 (1.97)	54,647 (1.55)
Hispanic/Latino or Hispanic and > 1 race	971,934 (40.78)	4,986,916 (26.90)	150,455 (26.97)	659,878 (18.69)
Native Hawaiian or other Pacific Islander	20,875 (0.88)	87,280 (0.47)	1,578 (0.28)	6,272 (0.18)
Non-Hispanic and > 1 race	13,354 (0.56)	150,640 (0.81)	2475 (0.44)	20,255 (0.57)
Unknown/Missing	251,473 (10.55)	1,527,267 (8.24)	101,052 (18.11)	476,928 (13.51)
Census region, # (%)				
Midwest	465,812 (19.55)	4,246,702 (22.90)	195,215 (34.99)	1,096,501 (31.06)
Northeast	366,972 (15.40)	2,744,037 (14.80)	126,581 (22.69)	584,428 (16.56)
South	499,261 (20.95)	7,865,663 (42.42)	88,218 (15.81)	1,317,225 (37.31)
West	1,051,225 (44.11)	3,684,341 (19.87)	147,898 (26.51)	531,867 (15.10)
Urban, # (%)	2,001,281 (84.04)	14,887,775 (80.35)	455,453 (81.73)	2,682,946 (76.06)
Distance to nearest health center, kilometers, mean (SD)	14.92 (20.86)	22.30 (25.54)	17.03 (26.80)	26.37 (30.06)
Temporary Assistance for Needy Families (TANF) eligible, # (%)	425,187 (17.84)	1,895,533 (10.22)	55,916 (10.02)	170,700 (4.84)
Medicaid eligibility, # (%)				
Child	2,291,934 (96.17)	17,542,569 (94.62)	530,403 (95.07)	3,224,660 (91.35)
Disabled	88,792 (3.73)	1,051,225 (5.10)	27,287 (2.89)	302,033 (8.56)
Demonstration projects	2,544 (0.11)	53,379 (0.29)	222 (0.04)	3,328 (0.09)
Eligible months, mean (SD)				
Total	11.41 (1.43)	11.38 (1.46)	11.43 (1.44)	11.46 (1.40)
Fee for service	3.33 (4.81)	2.81 (4.52)	11.43 (1.44)	11.46 (1.40)
Managed care	8.07 (4.97)	8.57 (4.67)	0 (0)	0 (0)
Chronic Disease Payment System Rx score, mean (SD)	0.99 (1.77)	1.17 (2.32)	1.11 (2.10)	1.36 (2.93)

### Demographics

Gender and age were similar in both groups. A higher proportion of HC patients were Hispanic/Latino (40.78% vs 26.90%) and a lower portion were White (23.27% vs 38.62%) compared to non-HC patients. Children receiving care at HCs were more likely to live in urban settings (84.04% vs 80.35%), live closer to the nearest HC (mean distance to nearest HC: 14.92 vs 22.30 km), and qualify for Temporary Assistance for Needy Families (TANF) (17.84% vs 10.22%) versus non-HC patients. Nearly all children were eligible for Medicaid based on parent income (child category). In terms of disease burden, HC patients had a lower CDPS score than non-HC patients (HC: 0.99 vs non-HC: 1.17), suggesting a lower level of chronic disease burden. Before weighting, all differences between groups were significant (*p* < 0.05) due to large population size. After weighting, HC and non-HC groups were equivalent on all characteristics (*p* > 0.05; Table 2).

### Primary analysis (Table 4)

**Table 4:** Utilization and quality of care among all children enrolled in Medicaid by primary care setting in the United States, 2012

Outcome	Crude^a^			Adjusted^a,b^		
	**HC (** ***n*** ** = 2,383,270)**	**Non-HC (** ***n*** ** = 18,540,743)**	**IRR / RR** ^**c**^ ** (95% CI)**	**HC (** ***n*** ** = 2,383,270)**	**non-HC (** ***n*** ** = 18,540,743)**	**IRR / RR** ^**c**^ ** (95% CI)**
**Utilization, per year – mean (95% CI)**						
Primary care visits	5.352	4.582	1.168	5.301	4.402	1.204
	(5.346,5.357)	(4.580,4.584)	(1.167,1.169)	(5.298,5.304)	(4.400,4.403)	(1.203,1.205)
Non-primary care outpatient visits	4.0581	5.8028	0.699	4.207	4.934	0.853
	(4.0455,4.0707)	(5.7964,5.8092)	(0.697,0.702)	(4.202,4.213)	(4.928,4.940)	(0.851,0.854)
ED visits	0.633	0.6756	0.937	0.634	0.6518	0.972
	(0.632,0.635)	(0.6751,0.6762)	(0.935,0.940)	(0.633,0.635)	(0.6513,0.6523)	(0.971,0.974)
Inpatient admissions	0.0668	0.0847	0.777	0.0659	0.0799	0.825
	(0.0654, 0.0663)	(0.0845, 0.0849)	(0.772,0.782)	(0.0656, 0.0663)	(0.0797, 0.0801)	(0.821,0.830)
Prescription claims	4.686	6.877	0.681	4.798	6.102	0.786
	(4.678,4.695)	(6.873,6.881)	(0.680,0.683)	(4.794,4.802)	(6.099,6.106)	(0.785,0.787)
**Utilization, per year – No. (%)**						
Children with 1 + ED visits	797,624 (33.47)	6,536,406 (35.25)	0.949 (0.948,0.951)	638,131 (33.91)	624,581 (33.19)	1.022 (1.019,1.027)
Children with 1 + inpatient admissions	109,022 (4.57)	1,063,004 (5.73)	0.7978 (0.793,0.803)	90,365 (4.80)	92,575 (4.91)	0.976 (0.967,0.985)
**Quality, per year – No. (%)**						
3–6 year old with well-child visit	395,919 (65.68)	2,742,950 (58.49)	1.123 (1.121,1.125)	310,621 (65.58)	270,629 (57.21)	1.146 (1.143,1.150)
12–17 year old with well-child visit	262,994 (49.51)	1,887,449 (46.90)	1.056 (1.053,1.059)	208,982 (49.91)	194,649 (46.48)	1.074 (1.069,1.078)
Children with ambulatory care sensitive ED visits	424,547 (17.81)	3,300,425 (17.80)	1.001 (0.998,1.004)	339,838 (18.06)	316,539 (16.82)	1.074 (1.069,1.078)
Children with ambulatory care sensitive hospitalizations	3,853 (0.16)	33,511 (0.18)	0.895 (0.865,0.925)	3,101 (0.16)	3,353 (0.18)	0.925 (0.881,0.971)

*Abbreviations: CI* confidence interval, *ED* emergency department, *HC* health center, *IRR* Incidence rate ratio, *RR* Relative risk ratio

^a^ The crude (unadjusted) estimates for the outcome variables were obtained for each group using generalized estimating equation models (with log link function and an appropriate distribution based on outcome type) that did not incorporate weights, while the adjusted estimates utilized overlap weights

^b^A total of 16,500 children were excluded from the adjusted analysis because data was missing for one or more of the characteristics used for matching

^c^IRR with 95% CI is presented for count variables with means. RR is presented for binary variables with numbers/percents

In terms of utilization, children who received the majority of their primary care at HCs had 20% more primary care visits per year than non-HC patients (IRR = 1.204, CI = 1.203–1.205). Outside of primary care, HC patients had 15% less outpatient visits than non-HC patients (IRR = 0.853, CI = 0.851–0.854). HC patients had 12% less prescription claims versus non-HC patients (IRR = 0.786, CI = 0.785–0.787).

For ED visits, we found some statistically significant differences between groups, but the magnitude was small compared to differences observed with other outcomes and direction differed depending on outcome construction (binary versus count). One-third of Medicaid-enrolled children had an ED visit that year. HC patients had slightly higher chance of having an ED visit on a relative basis (RR = 1.022, CI = 1.019–1.027), but the proportions of children with an ED visit were similar in absolute terms (HC 33.91% vs non-HC 33.19%). When examining total number of ED visits, HC patients had 3% fewer visits (IRR = 0.972, CI = 0.971–0.974).

In terms of hospitalizations, approximately 5% of Medicaid-enrolled children had an inpatient admission that year with HC patients having a 2% lower chance of hospitalization than non-HC patients (HC 4.80% vs non-HC 4.91%, RR = 0.976). Further, HC patients had 17% fewer inpatient admissions than non-HC patients (IRR = 0.825, CI = 0.821–0.830).

In terms of quality, a higher proportion of HC patients had well-child visits aligned with quality metrics than non-HC patients. For children 3–6 years, 65.58% of HC and 57.21% of non-HC patients had a well-child visit that year with a nearly 15% greater likelihood of well-child visits by children seen primarily at HCs versus non-HCs (RR = 1.146, CI = 1.143–1.150). Among children 12–17 years, 49.91% of HC patients had a well-child visit that year as compared to 46.48% of non-HC patients, making HC patients 7% more likely to do so (RR = 1.074, CI = 1.069–1.078). For ACS ED visits, 18.06% of children receiving primary care at HCs visited an ED for ACS conditions while 16.842 of non-HC children did, representing a 7% greater likelihood among HC patients versus non-HC (RR = 1.074, CI = 1.069–1.078). Few children had an ACS hospitalization, and HC patients were 7% less likely to do so than non-HC patients (0.16% vs 0.18%, RR = 0.925, CI = 0.881–0.971).

### Fee-for-service only (Table 5)

**Table 5:** Utilization, quality of care, and cost among children enrolled in fee-for-service Medicaid by primary care setting in the United States, 2012

Outcome	Crude^a^			Adjusted^a.b^		
	**HC (** ***n*** ** = 557,912)**	**Non-HC (** ***n*** ** = 3,530,021)**	**IRR / RR** ^**c**^ ** (95% CI)**	**HC (** ***n*** ** = 557,912)**	**Non-HC (** ***n*** ** = 3,530,021)**	**IRR / RR** ^**c**^ ** (95% CI)**
**Utilization, per year – mean (95% CI)**						
Primary care visits	4.569	4.879	0.937	4.576	4.614	0.992
	(4.559, 4.579)	(4.875, 4.883)	(0.934, 0.939)	(4.569,4.582)	(4.610,4.618)	(0.990, 0.993)
Non-primary care outpatient visits	5.866	9.790	0.599	6.1367	7.426	0.826
	(5.829, 5.903)	(9.766, 9.815)	(0.595, 0.603)	(6.118, 6.154)	(7.405, 7.447)	(0.823, 0.830)
ED visits	0.677	0.675	1.002	0.677	0.655	1.034
	(0.674, 0.680)	(0.674, 0.677)	(0.997, 1.008)	(0.675, 0.680)	(0.654, 0.656)	(1.030, 1.038)
Inpatient admissions	0.065	0.0769	0.844	0.065	0.0701	0.926
	(0.064, 0.066)	(0.0764, 0.0773)	(0.830, 0.857)	(0.064, 0.066)	(0.0697, 0.0705)	(0.915, 0.937)
Prescription claims	4.981	7.505	0.664	5.096	6.125	0.832
	(4.962, 5.000)	(7.494, 7.516)	(0.661, 0.666)	(5.086, 5.105)	(6.115, 6.134)	(0.830, 0.834)
**Utilization, per year – No. (%)**						
Children with 1 + ED visits	197,429 (35.39)	1,245,724 (35.29)	1.003 (0.999,1.007)	157,270 (35.53)	152,322 (34.42)	1.032 (1.027,1.038)
Children with 1 + inpatient admissions	23,299 (4.18)	172,777 (4.89)	0.853 (0.842,0.865)	18,884 (4.27)	18,667 (4.22)	1.012 (0.963,1.032)
**Quality, per year – No. (%)**						
3–6 year old with well-child visit	81,420 (59.71)	527,383 (61.02)	0.979 (0.974,0.983)	63,956 (59.51)	70,506 (65.61)	0.907 (0.901,0.913)
12–17 year old with well-child visit	67,243 (48.53)	434,738 (49.32)	0.984 (0.978,0.990)	53,431 (48.14)	58,490 (52.70)	0.914 (0.906,0.921)
Children with ambulatory care sensitive ED visits	100,391 (17.99)	572,228 (16.21)	1.110 (1.103,1.117)	79,546 (17.98)	71,149 (16.08)	1.118 (1.108,1.128)
Children with ambulatory care sensitive hospitalizations	707 (0.1267)	5,128 (0.1453)	0.872 (0.806,0.944)	560 (0.13)	608 (0.14)	0.921 (0.822,1.033)
**Cost, $ per year – mean (95% CI)**						
Total	2,644.49	3,808.78	0.694	2,716.08	2,955.23	0.919
	(2634.19, 2654.84)	(3,802.87, 3,814.69)	(0.691, 0.697)	(2,711.17, 2,720.99)	(2,949.90, 2,960.58)	(0.917, 0.921)
Primary care	794.65	574.75	1.383	791.03	545.10	1.451
	(792.37, 796.93)	(574.09, 575.40)	(1.378, 1.387)	(789.97, 792.10)	(544.37, 545.84)	(1.448, 1.454)
Non-primary care outpatient	869.52	1,576.03	0.552	902.62	1,199.34	0.753
	(864.15, 874.93)	(1,572.15, 1,579.92)	(0.548, 0.555)	(899.80, 905.48)	(1,195.60, 1,203.09)	(0.749, 0.756)
ED	173.92	184.06	0.945	174.88	175.20	0.998
	(172.97, 174.88)	(183.66, 184.46)	(0.939, 0.951)	(174.39, 175.36)	(174.72, 175.69)	(0.994, 1.002)
Inpatient	445.33	735.60	0.605	465.36	516.58	0.901
	(442.09, 448.60)	(733.37, 737.74)	(0.601,0.610)	(463.67, 467.06)	(514.70, 518.47)	(0.896, 0.906)
Pharmacy	366.43	743.69	0.493	387.56	524.38	0.739
	(364.62, 368.24)	(742.24, 745.16)	(0.490, 0.495)	(386.60, 388.52)	(523.08, 525.68)	(0.737, 0.742)

*Abbreviations: CI* confidence interval, *ED* emergency department, *HC* health center, *IRR* Incidence rate ratio, *RR* Relative risk ratio

^a^The crude (unadjusted) estimates for the outcome variables were obtained for each group using generalized estimating equation models (with log link function and an appropriate distribution based on outcome type) that did not incorporate weights, while the adjusted estimates utilized overlap weights

^b^A total of 5,634 children were excluded from the adjusted analysis because data was missing for one or more of the characteristics used for weighting

^c^Risk ratios are presented for binary variables with numbers/percents. IRR with 95% CI are presented for count variables with means

Characteristics of the fee-for-service population were largely similar to the pediatric Medicaid population in the primary analysis (Table 3). Key exceptions included that a lower proportion of children in the fee-for-service population were Hispanic/Latino, from the west, and TANF eligible as compared to all Medicaid. A higher proportion of fee-for-service patients were from the Midwest and qualified for Medicaid based on disability versus all Medicaid.

For utilization, children enrolled in fee-for-service Medicaid had a similar number of primary care visits in the year regardless of primary care location (IRR = 0.992, CI = 0.990–0.993). While this finding contrasts with the primary analysis, the remaining utilization outcomes aligned with results among all Medicaid-enrolled children. For quality, the proportions of children with well-child visits, ACS ED visits, and ACS hospitalizations were similar between fee-for-service and all Medicaid. Within the fee-for-service population, HC patients were 9–10% less likely to have a well-child visit than non-HC patients (3–6 years: RR = 0.907, CI = 0.901–0.913; 12–17 years: RR = 0.914, CI = 0.906–0.921).

In terms of expenditures, average total annual spending was $2,716.08 among HC patients and $2,955.23 among non-HC patients. Total expenditures were 8% lower for pediatric HC patients than non-HC (IRR = 0.919, CI = 0.917–0.921). Children who received the majority of their primary care at HCs had 45% higher primary care spending than those at non-HCs ($791.03 vs $545.10, IRR = 1.451, CI = 1.448–1.454). HC patients had 25% lower non-primary care outpatient spending ($902.62 vs $1,199.34, IRR = 0.753, CI = 0.749–0.756), similar ED spending ($174.88 vs $175.20, IRR = 0.998, CI = 0.994–1.002), 10% lower inpatient spending ($465.36 vs $516.58, IRR = 0.901, CI = 0.896–0.906), and 26% lower pharmacy spending ($387.60 vs $524.38, IRR = 0.739, CI = 0.737–0.742).

## Discussion

This is the first study to utilize nationwide claims data to evaluate healthcare utilization, quality, and spending among Medicaid-enrolled children who receive primary care at HCs versus non-HCs. Children who receive the majority of their primary care at HCs have similar or lower utilization (ED visits, hospitalizations) as well as similar quality of care with lower overall healthcare expenditures as compared to non-HCs. These findings suggest primary care delivery in HCs may be associated with a more cost-effective model of healthcare for children. 

Our results showed greater primary care utilization with lower non-primary care outpatient visits, lower prescription drug use, and lower inpatient admissions among HCs patients. Spending differences reinforced these utilization differences. The observation of more primary care use combined with less utilization of resource-intense services (e.g., hospitalization) is consistent with studies showing more primary care is associated with more effective care and lower spending [28–30]. HCs may provide a comprehensive model of primary care for children that may be associated with reduced use of more acute services which are considered “downstream” of primary care. This interpretation is consistent with the design of the HC program, which is constructed by-statute to align with the Medicaid population’s medically and socially complex needs. For example, HCs must be governed by a board of directors with a majority of representatives from their patient populations and maintain “enabling” services (e.g., translation, transportation) designed to increase access to care for safety-net populations.

Our findings do not demonstrate a clear pattern of lower ED use among HC patients, which appears inconsistent with the hypothesis that HC primary care reduces the need for higher acuity downstream services. A growing body of literature suggests ED care may serve as a complement to primary care, especially among those experiencing barriers to access, as compared to the traditional view of the ED serving as a substitute for primary care [10, 31]. For example, ED utilization may be greater if clinics have limited access to urgent care in the evenings and weekends, due to the hours of operation of HCs (e.g., no evening hours) and the guidance provided by voicemails and clinicians on call during evening and weekend hours (e.g., advise to go to ED if sick). An alternative explanation is that patients who are more engaged in primary care may also be empowered to use the ED as a point of access if primary or urgent care is unavailable.

Alternative rationales may explain the association we observe between health center use and utilization of non-primary care services. Differences in access to healthcare providers, hospitals, and/or pharmacies may lead HC patients to have lower utilization, for example limited access to subspecialists at HCs [32, 33]. Utilization may be related to factors which are not adequately accounted for by variables available in claims data. For example, patients’ social determinants of health outside of the healthcare system, such as transportation availability, [34, 35] parents’ employment status, ability to miss school/work, [36] or cultural factors [37].

When assessing the HC program, association between utilization and spending must be considered simultaneously with quality. Our well-child visit rates are comparable to national quality data [38]. We observed rates of well-child visits were greater among HC patients versus non-HC, indicating higher quality at HCs. ACS ED visits and hospitalizations had mixed findings between the two groups with less preventable inpatient hospitalizations but more preventable ED visits among HC patients. Overall, our quality measures provide mixed findings on the relative quality of care received by children in HCs versus non-HCs, which aligns with findings of prior studies examining the relationship between HCs and preventable ED visits or hospitalizations [39–41].

The analysis of expenditures focuses on the subset of children with fee-for-service Medicaid. While findings for some outcomes changed among the fee-for-service population compared to all-Medicaid population (e.g., primary care, ED), we found a pattern of lower expenditures in the HC group for all spending categories except primary care and emergency department. These results are consistent with survey-based studies showing children who receive primary care in HCs have significantly lower total annual spending versus non-HCs [4, 11].

As healthcare reform continues to unfold, our findings of lower spending and comparable quality among HC patients suggest the importance of this model of care, particularly for Medicaid populations. The shift to value-based payment requires that we uplift models of care that can optimize utilization, quality, and cost for diverse populations. The ongoing Coronavirus pandemic and long-standing structural racism have resulted in negative health, academic, and economic effects for children, particularly minority and low-income populations [42]. The results of this study suggest that it is critical for HC and Medicaid programs to continue to expand to serve as a true “safety-net” for populations in need. As the safety-net grows, policymakers will face increasing budgetary constraints, forcing decisions about where to direct limited healthcare dollars. Our findings suggest investments in HCs support an efficient model of care for children, laying the foundation for future generations.

This one-year, cross-sectional study is limited because it examines association but cannot show causation. Propensity score overlap weighting methods are used in this study to create comparable groups of HC and non-HC comparison children, however, we acknowledge that these methods can only control for differences in characteristics that are observable in our data. For example, the difference in illness burden between HC and non-HC patients may be greater than reflected by our risk adjustment covariate, which is based on observed differences in diagnoses found in the claims data. In addition, we use claims-based outcomes as proxies for clinical quality, which were based on utilization; however, these measures may provide limited insight into the true underlying quality differences that exist across settings (e.g., well-child visits as proxy for overall preventive care quality). Future studies should examine non-utilization based quality measures such as immunizations and patient-reported outcomes. Our study also has limitations with respect to insights on HC value. A full assessment of HC value requires comprehensive measurement of cost and benefit. However, claims data does not capture the federal support that HCs receive from grants programs (e.g., Sect. 330 Public Health Service Act) or other non-Medicaid support (e.g., federal government’s assumption of responsibility for malpractice settlement and judgment costs) [43]. Further, we cannot capture the differences and complexities in payment structures between HC and all possible comparison settings (e.g., higher indirect costs in academic settings). Our clinical quality measures also do not reflect the full scope of benefits that HCs provide to individuals or communities, for example potential reductions in health disparities or provision of jobs in underserved communities. Lastly, our study uses 2012 Medicaid data, the latest available nationwide when we began our study, and provides a benchmark prior to Medicaid expansion. Major policy changes, such as the Affordable Care Act and Medicaid managed care growth, may influence the generalizability of our results.

## Conclusions

For the pediatric population, receiving primary care at a health center was associated with higher primary care utilization and expenditures as well as similar or lower utilization and costs in non-primary care outpatient, ED, and inpatient care. Concurrently, quality of care was similar and total expenditures were lower. As the future of healthcare reform remains undecided, it is critical to recognize that HCs can be important parts of a high-value and efficient model of care for children. Investment in HCs has potential to support the health of children which is critical for them to live, learn, and play and thus fundamental to our country’s future.

### Supplementary Information

**Additional file 1:** **Appendix Table.** Utilization and quality of care among all children enrolled in Medicaid by primary care setting in the United States, 2012, based on the definition of children having 100% of primary care visits at health centers versus 100% at non-health centers.

## Data Availability

Data generated or analysed during this study are included in this published article. The data that support the findings of this study are available from the Centers for Medicare & Medicaid Services but restrictions apply to the availability of these data, which were used under license for the current study, and so are not publicly available. Data are however available from the corresponding author upon reasonable request and with permission of the Centers for Medicare & Medicaid Services.

## Abbreviations

ED: Emergency department
HC: Health center
HRSA: Health Resources and Services Administration

## References

[1] Health Resources and Services Administration. National Health Center Data. 2019. National Health Center Data. Available from: https://data.hrsa.gov/tools/data-reporting/program-data/national. Cited 2020.

[2] Health Resources and Services Administration. HRSA Health Center Program. 2020 Aug p. 2.

[3] National Association of Community Health Centers. Community Health Center Chartbook. 2020 p. 89. Available from: http://www.nachc.org/wp-content/uploads/2020/01/Chartbook-2020-Final.pdf. Cited 2020.

[4] Bruen BK, Ku L (2019). The Effects of Community Health Center Care on Medical Expenditures for Children and Adults: Propensity Score Analyses. J Ambulatory Care Manage.

[5] Health Resources and Services Administration. National Data: Selected Patient Characteristics. 2019. Available from: https://data.hrsa.gov/tools/data-reporting/program-data/national/table?tableName=4&year=2019. Cited 2020.

[6] Health Resources and Services Administration. The Affordable Care Act and Health Centers. 2012. Available from: https://www.hrsa.gov/sites/default/files/about/news/2012tables/healthcentersacafactsheet.pdf. Cited 2020.

[7] Shin P, Sharac J, Rosenbaum S (2015). Community Health Centers And Medicaid At 50: An Enduring Relationship Essential For Health System Transformation. Health Aff (Millwood).

[8] United States Government Accountability Office. Health Centers: Trends in Revenue and Grants Supported by the Community Health Center Fund. 2019. Report No.: GAO-19–496. Available from: https://www.gao.gov/assets/700/699394.pdf. Cited 2020.

[9] Nath JB, Costigan S, Hsia RY (2016). Changes in Demographics of Patients Seen at Federally Qualified Health Centers, 2005–2014. JAMA Intern Med.

[10] Nath JB, Costigan S, Lin F, Vittinghoff E, Hsia RY (2016). Federally Qualified Health Center Access and Emergency Department Use Among Children. Pediatrics.

[11] Bruen B, Ku L (2017). Community Health Centers Reduce the Costs of Children’s Health Care. Geiger GibsonRCHN Community Health Found Res Collab.

[12] Goldman LE, Chu PW, Tran H, Romano MJ, Stafford RS (2012). Federally Qualified Health Centers and Private Practice Performance on Ambulatory Care Measures. Am J Prev Med.

[13] Centers for Medicare and Medicaid Services. Medicaid Analytic eXtract (MAX) General Information 2020. Available from: https://www.cms.gov/Research-Statistics-Data-and-Systems/Computer-Data-and-Systems/MedicaidDataSourcesGenInfo/MAXGeneralInformation. Cited 2020.

[14] Centers for Medicare and Medicaid Services. Children’s Health Care Quality Measures . 2020. Available from: https://www.medicaid.gov/medicaid/quality-of-care/performance-measurement/adult-and-child-health-care-quality-measures/childrens-health-care-quality-measures/index.html. Cited 2020.

[15] Ben-Isaac E, Schrager SM, Keefer M, Chen AY (2010). National Profile of Nonemergent Pediatric Emergency Department Visits. Pediatrics.

[16] Billings J, Parikh N, Mijanovich T. Emergency Department Use in New York City: A Substitute for Primary Care? Commonw Fund Issue Brief. 2000. 11665698

[17] Agency for Healthcare Research and Quality. Pediatric Quality Indicators Overview .2012. Available from: https://qualityindicators.ahrq.gov/Modules/pdi_resources.aspx. Cited 2020.

[18] Nocon RS, Lee SM, Sharma R, Ngo-Metzger Q, Mukamel DB, Gao Y (2016). Health Care Use and Spending for Medicaid Enrollees in Federally Qualified Health Centers Versus Other Primary Care Settings. Am J Public Health.

[19] Centers for Medicare and Medicaid Services. National Plan and Provider Enumeration System. Available from: https://nppes.cms.hhs.gov/#/. Cited 2020.

[20] United States Department of Agriculture Economic Research Service. Rural-Urban Commuting Area Codes .2020 Available from: https://www.ers.usda.gov/data-products/rural-urban-commuting-area-codes/. Cited 2020.

[21] Kronick R, Gilmer T, Dreyfus T, Lee L (2000). Improving health-based payment for Medicaid beneficiaries: CDPS. Health Care Financ Rev.

[22] Gilmer TP, Kronick RG (2011). Differences In The Volume Of Services And In Prices Drive Big Variations In Medicaid Spending Among US States And Regions. Health Aff (Millwood).

[23] Gilmer T, Kronick R, Fishman P, Ganiats TG (2001). The Medicaid Rx model: pharmacy-based risk adjustment for public programs. Med Care.

[24] Medicaid and CHIP Payment and Access Commission. Enrollment and spending on Medicaid managed care 2016 Available from: https://www.macpac.gov/subtopic/enrollment-and-spending-on-medicaid-managed-care/. Cited 2020.

[25] Byrd V, Dodd AH. Mathematica. Assessing the Usability of Encounter Data for Enrollees in Comprehensive Managed Care 2010–2011. Available from: https://www.mathematica.org/our-publications-and-findings/publications/assessing-the-usability-of-encounter-data-for-enrollees-in-comprehensive-managed-care-2010-2011. Cited 2020.

[26] Thomas  LE, Li F, Pencina MJ (2020). Overlap Weighting: A Propensity Score Method That Mimics Attributes of a Randomized Clinical Trial. JAMA.

[27] Li F, Morgan KL, Zaslavsky AM (2018). Balancing Covariates via Propensity Score Weighting. J Am Stat Assoc.

[28] Baicker K, Chandra A (2004). Medicare Spending, The Physician Workforce, And Beneficiaries’ Quality Of Care. Health Aff (Millwood).

[29] Starfield B, Shi L, Macinko J (2005). Contribution of Primary Care to Health Systems and Health. Milbank Q.

[30] Jabbarpour Y, Greiner A, Jetty A, Coffman M, Jose C, Petterson S, et al. Investing in Primary Care: A State-level Analysis Patient-Centered Primary Care Collaborative and Robert Graham Center; 2019 Available from: https://www.pcpcc.org/sites/default/files/resources/pcmh_evidence_report_2019_0.pdf.

[31] Callison K, Nguyen BT (2018). The Effect of Medicaid Physician Fee Increases on Health Care Access, Utilization, and Expenditures. Health Serv Res.

[32] Nakamura Y, Laberge M, Davis A, Formoso A (2019). Barriers and Strategies for Specialty Care Access through Federally Qualified Health Centers: A Scoping Review. J Health Care Poor Underserved.

[33] Shields AE, Finkelstein JA, Comstock C, Weiss KB (2002). Process of Care for Medicaid-Enrolled Children With Asthma: Served by Community Health Centers and Other Providers. Med Care.

[34] Syed ST, Gerber BS, Sharp LK (2013). Traveling Towards Disease: Transportation Barriers to Health Care Access. J Community Health.

[35] Wallace DJ, Ray KN, Degan A, Kurland K, Angus DC, Malinow A (2018). Transportation characteristics associated with non-arrivals to paediatric clinic appointments: a retrospective analysis of 51 580 scheduled visits. BMJ Qual Saf.

[36] Jhanjee I, Saxeena D, Arora J, Gjerdingen DK (2004). Parents’ Health and Demographic Characteristics Predict Noncompliance with Well-Child Visits. J Am Board Fam Pract.

[37] Nicholson E, McDonnell T, De Brún A, Barrett M, Bury G, Collins C (2020). Factors that influence family and parental preferences and decision making for unscheduled paediatric healthcare – systematic review. BMC Health Serv Res.

[38] US Department of Health and Human Services. 2013 Annual Report on the Quality of Care for Children in Medicaid and CHIP p. 112. Available from: https://www.medicaid.gov/medicaid/quality-of-care/downloads/2013-ann-sec-rept.pdf. Cited 2020.

[39] Probst JC, Laditka JN, Laditka SB. Association between community health center and rural health clinic presence and county-level hospitalization rates for ambulatory care sensitive conditions: an analysis across eight US states. BMC Health Serv Res 2009; 9(1). Available from: https://bmchealthservres.biomedcentral.com/articles/ 10.1186/1472-6963-9-134. Cited 2019. PMC2727502 19646234

[40] Rust G, Baltrus P, Ye J, Daniels E, Quarshie A, Boumbulian P (2009). Presence of a Community Health Center and Uninsured Emergency Department Visit Rates in Rural Counties. J Rural Health.

[41] Falik M, Needleman J, Wells BL, Korb J. Ambulatory Care Sensitive Hospitalizations and Emergency VisitsExperiences of Medicaid Patients Using Federally Qualified Health Centers. Med Care. 2001;39(6):551–61. 10.1097/00005650-200106000-00004 11404640

[42] Kim L, Whitaker M, O’Halloran A, Kambhampati A, Chai SJ, Reingold A (2020). Hospitalization Rates and Characteristics of Children Aged <18 Years Hospitalized with Laboratory-Confirmed COVID-19 — COVID-NET, 14 States, March 1–July 25, 2020. Morb Mortal Wkly Rep.

[43] Takach M, Grossmann L, Hess C. Community Health Centers and State Health Policy: A Primer for Policymakers. National Academy of State Health Policy; 2012 p. 42. Available from: https://www.nashp.org/wp-content/uploads/sites/default/files/chc.primer.2012.2.pdf. Cited 2020.

