# Supplementary material for: Utilization, quality, and spending for pediatric Medicaid enrollees with primary care in health centers vs non-health centers
Source: BMC Pediatr. 2024 Feb 8;24:100. doi: 10.1186/s12887-024-04547-y (PMC10851548; doi:10.1186/s12887-024-04547-y)
Supplement: Supplementary file 1 — Additional file 1: Appendix Table. Utilization and quality of care among all children enrolled in Medicaid by primary care setting in the United States, 2012, based on the definition of children having 100% of primary care visits at health centers versus 100% at non-health centers. [file 12887_2024_4547_MOESM1_ESM.docx]

| \| **Appendix Table: Utilization and quality of care among all children enrolled in Medicaid by primary care setting in the United States, 2012, based on the definition of children having 100% of primary care visits at health centers versus 100% at non-health centers** \| \| \| \| \| \| \| \| \| --- \| --- \| --- \| --- \| --- \| --- \| --- \| --- \| \|  \| **Crude^a^** \| \| \| \| **Adjusted**^a,b^ \| \| \| \| **Outcome** \| **HC (n=2,383,270)** \| \| **Non-HC (n=18,540,743)** \| **IRR / RR^c^**  **(95% CI)** \| **HC (n=2,383,270)** \| **non-HC (n=18,540,743)** \| **IRR / RR^c^**  **(95% CI)** \| \| **Utilization, per year – mean (95% CI)** \| \| \| \| \| \| \| \| \| Primary care visits \| \| 3.706 \| 4.478 \| 0.828 \| 3.704 \| 4.159 \| 0.8907 \| \| (3.701,3.710) \| (4.477,4.480) \| (0.826,0.829) \| (3.701,3.707) \| (4.157,4.160) \| (0.8899,0.8914) \| \| Non-primary care outpatient visits \| \| 3.399 \| 5.7226 \| 0.594 \| 3.476^d^ \| 4.258^d^ \| 0.817^d^ \| \| (3.387,3.41) \| (5.7161,5.729) \| (0.592,0.596) \| (3.467,3.486) \| (4.247,4.268) \| (0.814,0.820) \| \| ED visits \| \| 0.557 \| 0.6647 \| 0.838 \| 0.556 \| 0.6243 \| 0.891 \| \| (0.555,0.558) \| (0.6641,0.6652) \| (0.835,0.840) \| (0.555,0.558) \| (0.6238,0.6247) \| (0.889,0.893) \| \| Inpatient admissions \| \| 0.0517 \| 0.0828 \| 0.624 \| 0.0516 \| 0.0731 \| 0.706 \| \| (0.0512,0.0521) \| (0.0826,0.0830) \| (0.618,0.629) \| (0.0513,0.0520) \| (0.0729,0.0732) \| (0.701,0.711) \| \| Prescription claims \| \| 3.758 \| 6.804 \| 0.552 \| 3.774 \| 5.686 \| 0.664 \| \| (3.750,3.7661) \| (6.800,6.809) \| (0.551,0.554) \| (3.771,3.778) \| (5.683,5.689) \| (0.663,0.665) \| \| **Utilization, per year – No. (%)** \| \| \| \| \| \| \| \| \| Children with 1+ ED visits \| \| 525,137 (30.65) \| 6,170,167 (34.87) \| 0.879 (0.877,0.881) \| 435,520 (30.81) \| 446,435 (31.48) \| 0.976 (0.972,0.979) \| \| Children with 1+ inpatient admissions \| \| 61,443 (3.59) \| 993,025 (5.61) \| 0.639 (0.634,0.644) \| 52,451 (3.71) \| 58,028 (4.11) \| 0.904 (0.893,0.914) \| \| **Quality, per year – No. (%)** \| \| \| \| \| \| \| \| \| 3-6 year old with  well-child visit \| \| 283,946 (64.85) \| 2,600,631 (58.12) \| 1.116  (1.113,1.118) \| 230,402 (64.29) \| 201,581 (56.25) \| 1.143 (1.139,1.447) \| \| 12-17 year old with  well-child visit \| \| 186,252 (49.15) \| 1,783,782 (46.58) \| 1.055  (1.051,1.059) \| 152,821 (48.79) \| 145,727 (46.53) \| 1.049  (1.043,1.054) \| \| Children with ambulatory care sensitive ED visits \| \| 282,268 (16.47) \| 3,107,845 (17.56) \| 0.938 (0.935,0.941) \| 233,769 (16.54) \| 226,247 (16.01) \| 1.033 (1.028,1.039) \| \| Children with ambulatory care sensitive hospitalizations \| \| 2,181 (0.13) \| 31,312 (0.18) \| 0.719 (0.689,0.751) \| 1,829 (0.13) \| 2,121 (0.15) \| 0.862 (0.810,0.918) \| \| Abbreviations: CI=confidence interval, ED=emergency department, HC=health center, IRR=Incidence rate ratio, RR=Relative risk ratio  ^a^ The crude (unadjusted) estimates for the outcome variables were obtained for each group using generalized estimating equation models (with log link function and an appropriate distribution based on outcome type) that did not incorporate weights, while the adjusted estimates utilized overlap weights.  ^b^A total of 16,500 children were excluded from adjusted analysis because data was missing for one or more of the characteristics used for matching.  **^c^**IRR with 95% CI is presented for count variables with means. RR is presented for binary variables with numbers/percents.  ^d^The outcome of non-primary care outpatient visits cannot be estimated by the weighted generalized linear model (GLM) with negative binomial distribution because the dispersion parameter was too large to converge. Therefore, we used the weighted GLM with the scaled Poisson distribution, where the overdispersion parameter was scaled and estimated using the Pearson statistic. This approach is based on direct guidance from SAS (<https://support.sas.com/kb/56/549.html)> and also used in the following article: Dewi Retno Sari Saputro, Ade Susanti, Nafisa Berliana Indah Pratiwi; The handling of overdispersion on Poisson regression model with the generalized Poisson regression model. *AIP Conf. Proc.* 8 February 2021; 2326 (1): 020026. <https://doi.org/10.1063/5.0040330.> \| \| \| \| \| \| \| \| |  |
| --- | --- | --- | --- | --- | --- | --- | --- | --- | --- | --- | --- | --- | --- | --- | --- | --- | --- | --- | --- | --- | --- | --- | --- | --- | --- | --- | --- | --- | --- | --- | --- | --- | --- | --- | --- | --- | --- | --- | --- | --- | --- | --- | --- | --- | --- | --- | --- | --- | --- | --- | --- | --- | --- | --- | --- | --- | --- | --- | --- | --- | --- | --- | --- | --- | --- | --- | --- | --- | --- | --- | --- | --- | --- | --- | --- | --- | --- | --- | --- | --- | --- | --- | --- | --- | --- | --- | --- | --- | --- | --- | --- | --- | --- | --- | --- | --- | --- | --- | --- | --- | --- | --- | --- | --- | --- | --- | --- | --- | --- | --- | --- | --- | --- | --- | --- | --- | --- | --- | --- | --- | --- | --- | --- | --- | --- | --- | --- | --- | --- | --- | --- | --- | --- | --- | --- | --- | --- | --- | --- | --- | --- | --- | --- | --- | --- | --- | --- | --- | --- | --- | --- | --- | --- | --- | --- | --- | --- | --- | --- | --- | --- | --- | --- | --- | --- | --- | --- | --- | --- | --- | --- | --- | --- | --- | --- |
